# Turning the Tide against Antibiotic Resistance by Evaluating Novel, Halogenated Phenazine, Quinoline, and NH125 Compounds against *Ureaplasma* Species Clinical Isolates and *Mycoplasma* Type Strains

**DOI:** 10.1128/AAC.02265-18

**Published:** 2019-02-26

**Authors:** Marissa A. Valentine-King, Katherine Cisneros, Margaret O. James, Robert W. Huigens, Mary B. Brown

**Affiliations:** a Department of Environmental and Global Health, College of Public Health and Health Professions, University of Florida, Gainesville, Florida, USA; b Department of Medicinal Chemistry, College of Pharmacy, University of Florida, Gainesville, Florida, USA; c Department of Infectious Diseases and Immunology, College of Veterinary Medicine, University of Florida, Gainesville, Florida, USA

**Keywords:** *Mycoplasma genitalium*, *Mycoplasma pneumoniae*, NH125 analogues, *Ureaplasma*, drug evaluation, nitroxoline, quinoline

## Abstract

Escalating levels of antibiotic resistance in mycoplasmas, particularly macrolide resistance in Mycoplasma pneumoniae and M. genitalium, have narrowed our antibiotic arsenal. Further, mycoplasmas lack a cell wall and do not synthesize folic acid, rendering common antibiotics, such as beta-lactams, vancomycin, sulfonamides, and trimethoprim, of no value. To address this shortage, we screened nitroxoline, triclosan, and a library of 20 novel, halogenated phenazine, quinoline, and NH125 analogues against *Ureaplasma* species and M. hominis clinical isolates from urine. We tested a subset of these compounds (*n* = 9) against four mycoplasma type strains (M. pneumoniae, M. genitalium, M. hominis, and Ureaplasma urealyticum) using a validated broth microdilution or agar dilution method. Among 72 *Ureaplasma* species clinical isolates, nitroxoline proved most effective (MIC_90_, 6.25 µM), followed by an *N*-arylated NH125 analogue (MIC_90_, 12.5 µM). NH125 and its analogue had significantly higher MICs against U. urealyticum isolates than against U. parvum isolates, whereas nitroxoline did not. Nitroxoline exhibited bactericidal activity against U. parvum isolates but bacteriostatic activity against the majority of U. urealyticum isolates. Among the type strains, the compounds had the greatest activity against M. pneumoniae and M. genitalium, with 8 (80%) and 5 (71.4%) isolates demonstrating MICs of ≤12.5 µM, respectively. Triclosan also exhibited lower MICs against M. pneumoniae and M. genitalium. Overall, we identified a promising range of quinoline, halogenated phenazine, and NH125 compounds that showed effectiveness against M. pneumoniae and M. genitalium and found that nitroxoline, approved for use outside the United States for the treatment of urinary tract infections, and an *N*-arylated NH125 analogue demonstrated low MICs against *Ureaplasma* species isolates.

## Abstract (precis)

Escalating levels of antibiotic resistance in mycoplasmas, particularly macrolide resistance in Mycoplasma pneumoniae and M. genitalium, have narrowed our antibiotic arsenal. Further, mycoplasmas lack a cell wall and do not synthesize folic acid, rendering common antibiotics, such as beta-lactams, vancomycin, sulfonamides, and trimethoprim, of no value.

## INTRODUCTION

Annually in the United States, antibiotic-resistant organisms cause an estimated 2 million illnesses and 20,000 deaths (1). Reports of antibiotic resistance extend to include mycoplasmas, which are fastidious bacterial organisms that cause urogenital or respiratory tract infections in pediatric and adult populations (2–4). As mycoplasmas lack a cell wall and do not synthesize folic acid, they are refractory to many widely used antibiotics, such as beta-lactams, vancomycin, sulfonamides, and trimethoprim (2). Therefore, this restricts treatment options to drugs that interfere with DNA replication or protein synthesis (2). With the confluence of emerging antibiotic resistance in mycoplasmas and their innate resistance to many commonly prescribed pharmaceuticals, a great impetus exists to identify novel drug classes capable of treating infections caused by these pathogens.

Mycoplasma pneumoniae causes an estimated 2 million cases of pneumonia in the United States annually, and the highest number of cases cluster in school-aged children and adolescents (3). Current treatment options in the pediatric population center on macrolides, as quinolones are not recommended as first-line therapy (5) and tetracycline use is limited to patients older than age 8 years (6). In Asia, macrolide-resistant M. pneumoniae (MRMP) strains have been reported to be widespread. In Beijing, China, 90% of adult and pediatric M. pneumoniae respiratory isolates collected between 2008 and 2012 were macrolide resistant (7), and macrolide resistance was found in 50% to 93% of M. pneumoniae isolates from specimens collected across seven regions in Japan during the 2010 to 2012 epidemic (8). Conversely, within that same time period in the United States, the MRMP prevalence ranged from 10 to 13.2% (9, 10). High levels of MRMP in Japan have been attributed to the selective pressure created from elevated macrolide prescribing (11). As macrolide prescribing in the United States is on the rise and mirrors the proportion seen in some Japanese reports (12, 13), researchers emphasize the need to revisit existing drug classes, as well as identify novel ones, to treat emerging MRMP infections.

New treatment modalities are also desperately needed for M. genitalium, a sexually transmitted pathogen that is most known for causing nongonococcal urethritis (NGU) in men (14) and that is associated with cervicitis, pelvic inflammatory disease, preterm birth, and spontaneous abortion in women (4). Globally, extremely high levels of mutations conferring macrolide resistance in M. genitalium (MRMG) have been found in Greenland (100%), New Zealand (72% to 77%), and Australia (36% to 79.4%), whereas moderate to high levels were detected in Spain (35%), Japan (29% to 47%), Denmark (38% to 57%), Germany (52.6%), Norway (56.4%), Canada (47% to 58%), the United States (48 to 80%), and England (41% to 82%) (15–37). Lower levels of MRMG have been reported in Russia (4.6%), Belgium (6.5%), France (8.3% to 11.3%), Africa (9.8% to 14.6%), Estonia (10%), Sweden (17.6% to 18.1%), and the Netherlands (21%) (38–46). A few studies found indications that the rate of resistance may be rising over time, evidenced by a significant increase in azithromycin treatment failures (47) and an increase in mutations associated with macrolide or quinolone resistance compared to earlier periods (26, 27, 48). More worrisome is the finding that as azithromycin and quinolones serve as the first- and second-line anti-M. genitalium treatment regimens in the United States, respectively, the emergence of quinolone resistance-associated mutations (QRMs) and mutations conferring dual resistance to both macrolides and quinolones has further depleted treatment options (49). The levels of QRMs in Japanese M. genitalium specimens appear to be rising and occurred in 53% of samples collected from 2010 to 2017, with dual resistance being reported in 25% of M. genitalium isolates in specimens from separate populations of Japanese men with urethritis and female sex workers (26–28). Studies in Australasia have also reported concerning levels of QRMs among 23.3% of samples from New Zealand, 15.4% from Sydney, Australia, and 13.6% from Melbourne, Australia, wherein 12 (8.6%) samples in the last study had mutations conferring dual resistance (16, 19, 50). In two studies based in Birmingham, AL, QRMs and mutations conferring dual resistance were detected in nearly a third of specimens from a population of men who have sex with men and roughly 11% of African-American heterosexual partners (33, 34). In three separate Canadian studies, QRMs in M. genitalium were found in about 2% of isolates from specimens from women, 12% of isolates from specimens from men, and 20% of isolates from samples derived from a population comprised mostly of men (29–31). These multidrug-resistant organisms limit physicians to doxycycline, which exhibits low cure rates, ranging from 30% to 45%, or to imported drugs that require special permits (51–53). Europe and Australia have responded to rising resistance by developing resistance-guided prescribing recommendations (54, 55). Australia’s recently released guidelines now recommend pretreating M. genitalium infections with doxycycline, as it reduces the organism load and lacks a penchant for inducing resistance, followed by treating susceptible infections with either azithromycin or moxifloxacin (55).

In the United States, concerning levels of tetracycline resistance have been reported in *Ureaplasma* spp., a type of mycoplasma most recognized for its role in negative reproductive outcomes and infections in neonates (2, 56–59). Two separate studies detected tetracycline resistance in 33% and 34% of *Ureaplasma* species isolates (60, 61), and a separate report identified the *tet*(M) gene, which confers tetracycline resistance and which was present in 45% of clinical isolates sourced from distinct geographic areas (2). However, lower levels of tetracycline resistance (range, 0.4% to 1.4%) and quinolone resistance (range, 1.4% to 6%) have been described in the United States (62, 63). In contrast, levofloxacin resistance was detected in 57% and in 75% of isolates from a Japanese study and a Chinese study, respectively (64, 65). Besides these notable studies, Beeton and Spiller have provided a comprehensive review of recent studies evaluating antibiotic resistance among *Ureaplasma* species isolates, as well as discussions examining the shortcomings of different testing kits that may influence the comparability of results across studies (66).

Few studies that abide by validated methods have evaluated antibiotic resistance in M. hominis, a mycoplasma associated with pelvic inflammatory disease, pyelonephritis, and other reproductive sequelae (2). Studies in the United States and Germany have demonstrated increases in clindamycin and tetracycline resistance over time. In the U.S. study, clindamycin and tetracycline resistance levels increased from 2% and 7%, respectively, in the late 1970s to 10% and 27%, respectively, a decade later (67). Similarly, Krausse and Schubert measured tetracycline resistance in 2% of isolates from 1983, a rate which then increased to 17% from 1997 to 2004 (68). Clindamycin resistance also increased over the same time frame from 0% to 11% (68). In French studies, Degrange et al. found tetracycline resistance in nearly 19% of isolates in the early 2000s (69), while Meygret et al. found resistance in approximately 15% of isolates collected from 2010 to 2015 (90). A recent U.S. study that tested 10 M. hominis isolates from women with first-time urinary tract infection (UTI) found no resistance to tetracycline, clindamycin, or levofloxacin (62).

**FIG 1:**
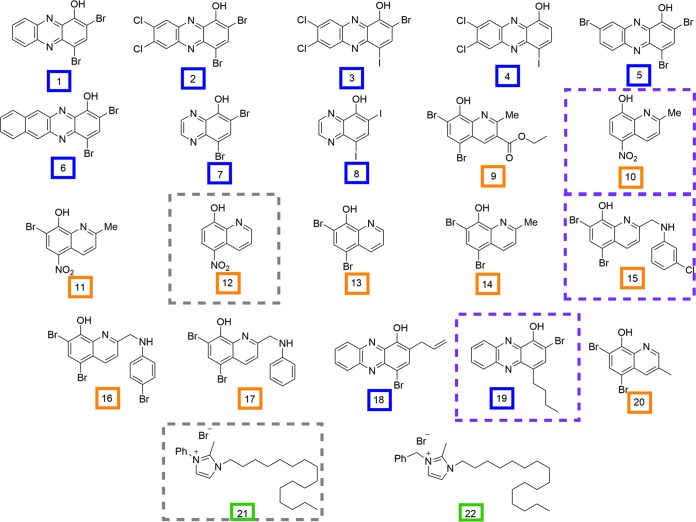
Compounds synthesized by the R. W. Huigens III lab. Halogenated phenazine, quinoline, and NH125 analogues have blue, orange, and green boxes around the compound numbers, respectively, designating each class. Compounds with a gray, dashed box represent those that demonstrated efficacious MICs against *Ureaplasma* species clinical isolates and M. genitalium and M. pneumoniae type strains. Those highlighted with a dashed purple box had efficacious MICs against both M. genitalium and M. pneumoniae, mycoplasmas with high levels of macrolide resistance in recent years. Me, methyl; Ph, phenyl.

In order to expand our existing arsenal of antibiotics, we evaluated the MICs of 22 halogenated phenazine, quinoline, and NH125 analogues (Fig. 1) and triclosan against the following human mycoplasmas: M. genitalium, M. hominis, M. pneumoniae, and *Ureaplasma* spp. These analogue libraries were synthesized by substituting various halogen or methyl groups at critical positions along the base structure of a previously efficacious, representative compound for each class (70–72). This led to an expanded library of halogenated phenazine, quinoline, and NH125 analogues that had, in some cases, MICs comparable to or more efficacious than those of leading antimicrobials used to treat methicillin-resistant Staphylococcus aureus (MRSA), methicillin-resistant S. epidermidis (MRSE), and vancomycin-resistant Enterococcus faecium (VRE) (70–72). Mechanistically, past investigations have accumulated evidence suggesting that the NH125 analogues exert their effects through bacterial membrane destruction (73), whereas the quinolines and halogenated phenazine compounds appear to work through a non-membrane-destroying, metal(II)-dependent mechanism (70, 74–76). As mycoplasmas lack a cell wall, which leaves an exposed cell membrane, we hypothesize that NH125 analogues will result in low MICs against human mycoplasmas.

## RESULTS

### Clinical isolate MICs.

**FIG 2:**
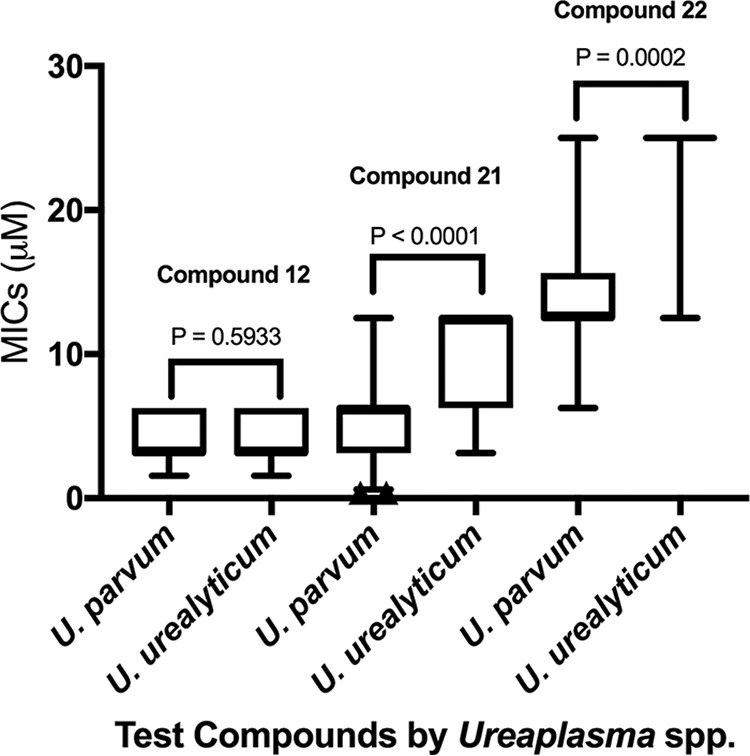
Box plots displaying MIC comparisons between *Ureaplasma* spp. for compounds 12, 21, and 22. To depict comparisons for antimicrobial compound 22, MICs of >12.5 µM were designated 25 µM for the purposes of this figure only. Whiskers depict 5th and 95th percentiles.

The majority of compounds 1 to 22 had MICs of >12.5 µM against the screened, *Ureaplasma* species clinical isolates (Table 1), with the exception of compounds 12 (nitroxoline), 10 (a quinoline structurally similar to nitroxoline), 21 (an NH125 analogue), and 22 (NH125). Nitroxoline had the lowest MIC_50_ (3.13 µM) and MIC_90_ (6.25 µM) values against *Ureaplasma* spp. Interestingly, *Ureaplasma* species isolates that had MICs of ≤12.5 µM to compound 10 also had significantly lower MIC values to nitroxoline (*P* = 2.89 × 10^−5^). Compound 21 had the second lowest MIC_50_ and MIC_90_ values among the *Ureaplasma* species isolates. *Ureaplasma* species isolates with MICs of ≤12.5 µM to NH125 also had significantly lower MICs to compound 21 (*P* = 4.82 × 10^−7^). NH125 had MICs of ≤12.5 µM in 65.3% (*n* = 47) of isolates, while compound 10 had MICs of ≤12.5 µM in 50% (*n* = 17) of the screened isolates (*n* = 34). No significant differences existed when comparing MIC values for compound 10 and nitroxoline between Ureaplasma urealyticum and U. parvum isolates; however, compound 21 and its parent compound, NH125, had significantly lower (*P* < 0.0001) MICs against U. parvum isolates than against U. urealyticum isolates (Fig. 2). The levofloxacin- and tetracycline-resistant U. parvum isolates had MICs that fell in line with the MICs displayed by sensitive U. parvum isolates. The MICs of nitroxoline and compound 21 were 6.25 µM for both the levofloxacin- and tetracycline-resistant U. parvum isolates, which matches the MIC_90_ and MIC_50_ of these compounds, respectively. The MIC of compound 22 for both sets of isolates was 12.5 µM, which was the MIC_50_ for U. parvum isolates. In general, the MIC values of the compounds for U. urealyticum ATCC 33175 (Table 2) fell within the range of MICs observed for all clinical isolates, with the exception of compound 21 (MIC > 25 µM). Triclosan had MICs of >120 µM against the subset of *Ureaplasma* species and M. hominis clinical isolates and type strains tested (data not pictured). Initially, when screening nitroxoline against a subset of M. hominis isolates, we obtained MIC values of ≤12.5 µM against the type strain (6.25 µM) and two clinical isolates (12.5 µM). However, we could not reproduce these results and found that nitroxoline had a MIC of 50 µM against four clinical isolates and the type strain, while the remaining isolates had MICs of >50 µM.

**TABLE 1:** Summary of MIC results for 72 *Ureaplasma* species clinical isolates for test compounds with MICs of ≤12.5 µM for >20% of the screened isolates

Isolate and test compound^*a*^ (no. of isolates)	MIC^*b*^			No. (%) of resistant isolates^*c*^
	Range	50%	90%	
U. parvum (*n* = 59)				
Compound 10 (*n* = 26)	12.5 to >12.5	12.5	>12.5	NA
Compound 12 (*n* = 59)	1.56 to 6.25	3.13	6.25	NA
Compound 21 (*n* = 59)	0.39 to 12.5	6.25	12.5	NA
Compound 22 (*n* = 59)	6.25 to >12.5	12.5	>12.5	NA
Levofloxacin (*n* = 59)^*d*^	≤0.25 to 4	0.5	1	1 (1.7)
Tetracycline (*n* = 59)^*d*^	≤0.25 to 8	≤0.25	0.5	1 (1.7)
U. urealyticum (*n* = 13)				
Compound 10 (*n* = 8)	12.5 to >12.5	12.5	>12.5	NA
Compound 12 (*n* = 13)	1.56 to 6.25	3.13	6.25	NA
Compound 21 (*n* = 13)	3.13 to 12.5	12.5	12.5	NA
Compound 22 (*n* = 13)	12.5 to >12.5	>12.5	>12.5	NA
Levofloxacin (*n* = 13)^*d*^	0.5 to 1	1	1	0
Tetracycline (*n* = 13)^*d*^	≤0.25 to 1	1	1	0

a Compound 12 is also known as nitroxoline, and compound 22 is known as NH125.

b MICs are in micromolar for compounds 10, 12, 21, and 22 and micrograms per milliliter for levofloxacin and tetracycline.

c *Ureaplasma* spp. determined by using CLSI interpretive guidelines. *Ureaplasma* species breakpoints were as follows: levofloxacin, ≥4 µg/ml; tetracycline, ≥2 µg/ml. NA, not applicable.

d MIC values for levofloxacin and tetracycline were reported by Valentine-King and Brown (62).

**TABLE 2:** MIC results for a subset of the test compounds and triclosan against human mycoplasma type strains M. genitalium, M. hominis ATCC 23411, and U. urealyticum ATCC 33175^*e*^

Organism	MIC (µM) for the following compounds:											QC drug MIC (µg/ml)		No. (%) of AMCs with MICs of ≤12.5 µM
	4	10	11	12	14	15	19	20	21	22	Triclosan	Levofloxacin	Tetracycline	
M. genitalium^*a*^	NA	12.5	NA	12.5	>12.5	12.5	12.5	>12.5	3.13	NA	120	1		5 (71.4)
M. pneumoniae^*b*^	>12.5	12.5	>12.5	12.5	6.25	6.25	6.25	2.35^*c*^	3.13	3.13	60	0.5		8 (80)
U. urealyticum^*b*^	>12.5	12.5	>12.5	3.13	>25	>25	>25	25	>25	NA	>120	1		2 (22.2)
M. hominis^*a*^	>12.5	>12.5	>12.5	50^*d*^	>12.5	>12.5	>12.5	>12.5	>12.5	>12.5	>120		1	(0)
														
No. (%) of AMCs with MICs of ≤12.5 µM	0 (0)	3 (75)	0 (0)	3 (75)	1 (25)	2 (50)	2 (50)	1 (25)	2 (50)	1 (50)				

a Tested compounds against this organism in doubling dilutions ranging from 12.5 µM to 1.56 µM.

b Tested compounds against this organism in doubling dilutions ranging from 25 µM to 3.13 µM, except for compounds 4 and 11, which were tested in doubling dilutions from 12.5 µM to 1.56 µM.

c Midpoint value of the MIC range from two independent tests.

d Tested in doubling dilutions ranging from 50 µM to 6.25 µM.

e NA, not applicable (not tested); QC, quality control; AMC, antimicrobial compounds.

### M. genitalium and M. pneumoniae type strain MICs.

The highest efficacy was observed against M. pneumoniae and M. genitalium type strains, with 8 (80%) and 5 (71%) compounds having MICs of ≤12.5 µM against each one, respectively (Table 2). In particular, nitroxoline and compounds 10, 15, 19, and 21 had efficacious MICs against both mycoplasmas, and compound 21 had the lowest MIC against both (MIC, 3.13 µM). Compound 20 had the lowest MIC against M. pneumoniae (2.35 µM), followed by compound 21 and NH125 (MICs, 3.13 µM) and then compounds 14, 15, and 19 (MICs, 6.25 µM). When comparing the MIC values of the same compounds between the two type strains, M. pneumoniae displayed lower average MICs (6.25 µM) than M. genitalium (12.5 µM). Both M. pneumoniae and M. genitalium appeared to display similar sensitivities to the drugs among the drug classes. Further, triclosan had MICs of ≤120 µM to both M. genitalium and M. pneumoniae, which varied by only 1 doubling dilution factor (Table 2).

### MBC results.

We conducted minimum bactericidal concentration (MBC) assays using a subset of *Ureaplasma* species isolates and type strains and the compounds with efficacious MICs via broth microdilution. Nitroxoline demonstrated bactericidal effects against all U. parvum isolates tested (*n* = 10) but had mostly bacteriostatic effects against U. urealyticum (80%) isolates, one of which included the U. urealyticum ATCC 33175 type strain (Table 3). This difference in effect proved significantly different between species (*P* = 0.004). On the other hand, compound 21 (an *N*-arylated NH125 analogue) demonstrated bacteriostatic effects against all *Ureaplasma* spp. except for one U. parvum isolate (Table 3). Interestingly, this U. parvum isolate contained the *tet*(M) gene and displayed a tetracycline-resistant phenotype in a prior study (62).

**TABLE 3:** MBC results for compounds efficacious against a subset of *Ureaplasma* species clinical isolates and representative type strains

Compound, organism	No. of isolates for which MBC was:			No. (%) of isolates for which treatment was:			Range or avg (range) MBC (µM)
	2× MIC	4× MIC	>4× MIC	Bactericidal	Bacteriostatic	*P* value	
Compound 12							
U. urealyticum (*n* = 5)	1	0	4	1 (20)	4 (80)	0.004	6.25 to >12.5
U. parvum (*n* = 10)	7	3		10 (100)	0 (0)		12.5 (6.25 to 25)
Compound 21, *Ureaplasma* spp. (*n* = 9)	1	0	8	1 (11.1)	8 (88.9)		12.5 to >12.5

## DISCUSSION

Widespread MRMP in Asia (7, 8) coupled with notable levels of MRMG globally (15–37) have depleted first-line treatment options against these pathogenic organisms. This is reflected in the newest sexually transmitted disease treatment guidelines for M. genitalium in Australia and Europe (54, 55). Additionally, concerning levels of tetracycline resistance in *Ureaplasma* species isolates in the United States have been reported (2, 60, 61). With rising levels of antibiotic resistance eliminating the drug classes available for treating mycoplasma infections, identifying new drug classes serves a critical need. To address this emerging necessity, we selected a chemically diverse library of drug analogues with different modes of action and antibacterial profiles for this study. We screened our library, and two agents were found to be active against a subset of urinary *Ureaplasma* species clinical isolates. We then tested a subset of our library against four human mycoplasma type strains and identified a number of compounds with low MICs against both M. genitalium and M. pneumoniae.

Among the *Ureaplasma* species clinical isolates, we found that nitroxoline and the *N*-arylated NH125 analogue (compound 21) demonstrated the highest efficacy. NH125 was the third most effective compound, followed by compound 10, an analogue of nitroxoline. Nitroxoline MICs did not differ by species; therefore, this would give clinicians higher confidence in prescribing nitroxoline to cover infections caused by either *Ureaplasma* species (MIC_90_, 6.25 µM, or 1.19 µg/ml). Importantly, nitroxoline was efficacious against the *Ureaplasma* spp. and the M. genitalium type strain (MIC, 12.5 µM, or 2.38 µg/ml). Therefore, nitroxoline could provide coverage against most urogenital *Mollicutes*. Nitroxoline also was effective against the tetracycline- and levofloxacin-resistant U. parvum isolates. A French study (77) that tested nitroxoline against 30 U. urealyticum isolates reported a MIC_90_ value of 0.35 mg/liter (1.84 µM) and a MIC_90_ of 3 mg/liter (15.78 µM) against M. hominis isolates, both of which are lower than what we observed using Clinical and Laboratory Standards Institute (CLSI) standards (78). As their study occurred prior to publication of CLSI standards for evaluating MICs in *Mollicutes*, they did not standardize the inoculum used, which ranged from 1 to 10^5^ color-changing units (CCU)/ml for U. urealyticum and 10^1^ to 10^6^ CCU/ml for M. hominis (77). Thus, isolates tested with a CCU of 10^3^ or less could have had higher MICs.

As mycoplasmas evolved in a reductionist manner from Gram-positive bacteria, we compared our results for *Ureaplasma* spp. to those of prior studies evaluating nitroxoline and compound 21 efficacies against MRSA and MRSE clinical isolates and type strains. Nitroxoline performed substantially better against *Ureaplasma* species isolates (MIC range, 1.56 to 6.25 µM; average MIC, 4.21 µM) than against its Gram-positive relatives (MIC range, 9.38 to 25 µM; average MIC, 16.16 µM) (79). However, compound 21 fared slightly better against MRSA and MRSE strains (MIC range, 1.17 to 3.13 µM; average MIC, 1.95 µM) than against *Ureaplasma* species isolates (MIC range, 0.39 to 12.5 µM; average MIC, 6.19 µM) (71).

A previous study found that nitroxoline exhibited bactericidal effects against U. urealyticum and M. hominis isolates; however, this was prior to the separation of *Ureaplasma* into two distinct species (77). When evaluating whether the compounds exerted bactericidal or bacteriostatic effects against *Ureaplasma* spp., we found that nitroxoline was significantly more likely to exert a bactericidal effect against U. parvum isolates than against U. urealyticum isolates. The mechanism behind the activity of nitroxoline in planktonic and sessile biofilm communities appears to involve metal ion chelation (75, 76); however, differences in metal requirements between *Ureaplasma* species have not been explored, based on the current literature. Therefore, the significance behind this difference is unknown. Unlike nitroxoline, compound 21 exhibited bacteriostatic effects against the majority of clinical *Ureaplasma* species isolates (89%), independent of species. As previous studies have indicated that *N*-arylated NH125 compounds target cell membranes and demonstrate hemolysis at low doses (71), we expected compound 21 to produce bactericidal effects against *Ureaplasma* spp. However, in a previous study, compound 21 required a dose of 7.84 µM to lyse 50% of red blood cells (71). Thus, perhaps a higher dose is required to obtain a bactericidal effect in *Ureaplasma* spp., since compound 21 had a MIC_90_ of 12.5 µM.

Until now, only one study has evaluated triclosan efficacy against mycoplasmas, reporting MICs ranging from 16 µg/ml (55 µM) to 64 µg/ml (221 µM) when tested against mycoplasmas that infect food and fiber animals (80). Here we found evidence that triclosan had comparable, if not lower, MICs against two human mycoplasmas: M. pneumoniae (60 µM) and M. genitalium (120 µM). However, triclosan showed no effect against both *Ureaplasma* species and M. hominis clinical isolates and type strains.

In light of the high levels of MRMP and MRMG in recent years (7, 8, 15–37), it is encouraging that we identified a number of promising antimicrobial agents and preexisting compounds that demonstrated efficacy against M. genitalium and M. pneumoniae. We identified eight compounds (compounds 10, 12, 14, 15, and 19 to 22) that were efficacious against M. pneumoniae and five (compounds 10, 12, 15, 19, and 21) that were effective against M. genitalium. When comparing the MICs of efficacious compounds between M. pneumoniae and M. genitalium, M. pneumoniae had lower MICs across the board. M. pneumoniae was tested via broth microdilution, and M. genitalium was tested via agar dilution. This could explain the lower MICs seen in M. pneumoniae, as previous research has shown that agar dilution can increase the MIC by a factor of 4-fold (81). In general, both M. pneumoniae and M. genitalium were largely susceptible to the same compounds, which seems reasonable, as both cluster together phylogenetically (82). Furthermore, nitroxoline demonstrated low MICs against *Ureaplasma* spp. and is a preexisting compound approved for the treatment of UTIs in Europe. As compounds 12, 14, 15, 21, and 22 (71, 79) eradicated MRSA and MRSE biofilms, these compounds may also have the potential to disrupt mycoplasma biofilms, which have been detected in M. pneumoniae and *Ureaplasma* spp. (83–85). All of the compounds represent new classes of antimicrobials separate from those currently approved for use in the United States and could increase the limited antimicrobial arsenal available for these pathogens.

## MATERIALS AND METHODS

### Study description.

The clinical isolates of *Ureaplasma* spp. and M. hominis tested originated from a study that prospectively followed college-age women presenting with first-time urinary tract infection (UTI) at a student health care center in Florida between 2001 and 2006 (86). Clinical isolates of *Ureaplasma* spp. and M. hominis were obtained from direct culture of urine collected at either the initial UTI presentation or any recurrent UTI episode(s). An antibiogram characterizing the MIC_50_ and MIC_90_ values of a panel of antibiotics against the *Ureaplasma* species and M. hominis clinical isolates was previously published (62). Two U. parvum samples had resistant phenotypes: one for resistance to tetracycline and one for resistance to levofloxacin (62). In the current study, we evaluated the efficacy of NH125, an *N*-arylated NH125 analogue, 10 phenazine analogues, 10 quinoline analogues, and triclosan against these urinary clinical isolates using a previously validated broth microdilution or agar dilution method (78). The chemical structures for the parent compounds and analogues are shown in Fig. 1. We screened a subset of clinical isolates against all compounds. Compounds with efficacious MICs (≤12.5 µM) against the majority of clinical isolates were chosen for full testing against all 13 U. urealyticum and 59 U. parvum isolates. Nine compounds also were tested against three human mycoplasma type strains, M. hominis ATCC 23114, M. pneumoniae ATCC 29342, and U. urealyticum ATCC 33175, and one clinical isolate, M. genitalium (a gift from J. Baseman, University of Texas Health Sciences Center, San Antonio, TX).

M. pneumoniae and M. genitalium were grown in standard laboratory medium preparations of SP4 broth and agar supplemented with glucose (pH 7.6 to 7.8); for M. hominis, we used SP4 supplemented with l-arginine (pH 7.2 to 7.4); for *Ureaplasma* spp., we used 10B broth and A8 agar with a pH range of 5.9 to 6.1.

### Antimicrobial agents.

As an internal quality control, we included levofloxacin (U. urealyticum ATCC 33175 and M. pneumoniae ATCC 29342) and tetracycline or clindamycin (M. hominis ATCC 23114) to determine if the MICs fell within previously established ranges for these type strains (78). Although no quality control range exists for M. genitalium, we compared the MIC for levofloxacin against the values presented in previously published reports and used levofloxacin thereafter as an internal measure for quality control.

Levofloxacin powder was obtained from Sigma-Aldrich (St. Louis, MO, USA), clindamycin through Pfizer's Compound Transfer Program, and tetracycline through Cellgro (Herndon, VA, USA). When preparing stock solutions, we used established guidelines to adjust for drug purity (87) and used CLSI recommendations to dissolve and dilute the quality control drugs (88). The quality control drugs were tested in doubling dilutions that encompassed 1 dilution above and 1 dilution below the established quality control range. The 22 test compounds were provided at either 10 mM or 1 mM concentrations in dimethyl sulfoxide (DMSO) and were stored at room temperature with protection from light. Drugs were diluted in broth on the day of testing and tested within 6 months of receipt.

### MIC determination.

We followed a previously validated broth microdilution or agar dilution method to evaluate the MICs, as previously described (62, 78). For the broth microdilution assay, we used sterile 96-well plates wherein each row contained an antimicrobial agent in doubling dilutions ranging from 12.5 µM to 0.2 µM for clinical isolates and from 25 µM to 3.13 µM for each type strain, in duplicate. Duplicate growth controls, drug controls, solvent controls, and medium controls were set up for each drug and organism tested. A 1:10 dilution of DMSO served as the solvent control. Plates were inoculated with 175 µl of organism at between 10^4^ and 10^5^ CFU/ml. The organisms had been preincubated in broth either for 1 h for the *Ureaplasma* spp. or for 2 h for all other mycoplasmas tested. The plates were sealed with sterile acetate sealers, with the exception of those with M. pneumoniae, which were sealed per the protocol of Waites et al. (78), and incubated at 37°C in ambient air until the growth control displayed a distinct color change. We read the MIC as the lowest concentration of drug that inhibited any color change.

For organisms that did not show a distinct color change and for compounds that altered the broth color, we used a validated agar dilution method to evaluate drug MICs. Briefly, the method consisted of incorporating 600 µl of antibiotic within 5.4 ml of molten agar by adding the appropriate volume of stock antibiotic to yield concentrations spanning from 25 to 3.13 µM for each drug. We created solvent and growth control plates by mixing 5.4 ml of molten agar with 600 µl of a 1:10 DMSO solution and with 600 µl of filter-sterilized, double-distilled water, respectively. Following a 2-h preincubation period, we added three separate 20-µl drops of organism at 10^3^-, 10^4^-, and 10^5^-CFU/ml concentrations onto each agar plate. Using the organism dilution of between 10^4^ and 10^5^ CFU/ml, the MIC for each drug was read as the lowest antibiotic concentration that inhibited colony formation when the growth control plate exhibited colonies.

For compounds that had efficacious MICs against type strains, defined as a MIC of ≤12.5 µM, we performed a second, confirmatory MIC determination. For clinical isolates, we retested efficacious compounds against a subset of clinical isolates to confirm our results. We considered the MIC results valid only if the organism’s number of CFU ranged from 10^4^ to 10^5^ CFU/ml, which was confirmed on the day of testing.

### MBC determination.

We evaluated the MBCs of efficacious compounds for a subset of *Ureaplasma* species clinical isolates using a previously published method (89). The MBC assay called for transferring 30-µl aliquots directly from the MIC microtiter plate at 1, 2, and 4 times the drug MIC into culture tubes with 2.97 ml of fresh broth immediately following MIC interpretation. For the positive and negative controls, we transferred 30 µl from the growth control and 30 µl from the medium control into separate tubes with 2.97 ml of fresh broth. Following inoculation, all tubes were incubated at 37°C with ambient air for 7 days. Compounds were considered bactericidal if the lowest concentration that did not show growth was within one to four times the predetermined MIC level following incubation. Although the guidelines of the journal *Antimicrobial Agents and Chemotherapy* call for the use of an inoculum of >5 × 10^5^ CFU/ml for MBC assays with bacteria, the CLSI standardized assay for determining compound MICs in *Ureaplasma* spp. calls for the use of inoculum concentrations of between 10^4^ and 10^5^ CCU/ml or CFU/ml. Therefore, we conducted MBC assays using the inoculum range designated by the CLSI standardized assay.

### Statistical considerations.

Statistical comparisons of MICs between U. parvum and U. urealyticum isolates were conducted using either a Wilcoxon-Mann-Whitney test for MIC data in interval form (compounds 12 and 21) or a chi-square test for MICs characterized as either greater than or less than 12.5 µM (compounds 10 and 22). We used the Fisher exact test to compare differences in MBC outcomes between *Ureaplasma* spp. Statistical calculations were conducted using RStudio (version 1.0.136) software. A *P* value of <0.05 was considered statistically significant.
